# Characterization and pathogenesis of aerosolized eastern equine encephalitis in the common marmoset (*Callithrix jacchus*)

**DOI:** 10.1186/s12985-017-0687-7

**Published:** 2017-02-07

**Authors:** Aimee I. Porter, Rebecca A. Erwin-Cohen, Nancy Twenhafel, Taylor Chance, Steven B. Yee, Steven J. Kern, David Norwood, Laurie J. Hartman, Michael D. Parker, Pamela J. Glass, Luis DaSilva

**Affiliations:** 1 United States Army Medical Research Institute of Infectious Diseases (USAMRIID), Virology Division, Frederick, MD 21702 USA; 2 Pathology Division, Frederick, MD 21702 USA; 3 Center for Aerobiological Sciences, Frederick, MD 21702 USA; 4 Research Support Division, Frederick, MD 21702 USA; 5 Diagnostic Systems Division, Frederick, MD 21702 USA

**Keywords:** Animal model, Alphavirus, Immunity, Nonhuman primate, Pathogenesis

## Abstract

**Background:**

Licensed antiviral therapeutics and vaccines to protect against eastern equine encephalitis virus (EEEV) in humans currently do not exist. Animal models that faithfully recapitulate the clinical characteristics of human EEEV encephalitic disease, including fever, drowsiness, anorexia, and neurological signs such as seizures, are needed to satisfy requirements of the Food and Drug Administration (FDA) for clinical product licensing under the Animal Rule.

**Methods:**

In an effort to meet this requirement, we estimated the median lethal dose and described the pathogenesis of aerosolized EEEV in the common marmoset (*Callithrix jacchus*). Five marmosets were exposed to aerosolized EEEV FL93-939 in doses ranging from 2.4 × 10^1^ PFU to 7.95 × 10^5^ PFU.

**Results:**

The median lethal dose was estimated to be 2.05 × 10^2^ PFU. Lethality was observed as early as day 4 post-exposure in the highest-dosed marmoset but animals at lower inhaled doses had a protracted disease course where humane study endpoint was not met until as late as day 19 post-exposure. Clinical signs were observed as early as 3 to 4 days post-exposure, including fever, ruffled fur, decreased grooming, and leukocytosis. Clinical signs increased in severity as disease progressed to include decreased body weight, subdued behavior, tremors, and lack of balance. Fever was observed as early as day 2–3 post-exposure in the highest dose groups and hypothermia was observed in several cases as animals became moribund. Infectious virus was found in several key tissues, including brain, liver, kidney, and several lymph nodes. Clinical hematology results included early neutrophilia, lymphopenia, and thrombocytopenia. Key pathological changes included meningoencephalitis and retinitis. Immunohistochemical staining for viral antigen was positive in the brain, retina, and lymph nodes. More intense and widespread IHC labeling occurred with increased aerosol dose.

**Conclusion:**

We have estimated the medial lethal dose of aerosolized EEEV and described the pathology of clinical disease in the marmoset model. The results demonstrate that the marmoset is an animal model suitable for emulation of human EEEV disease in the development of medical countermeasures.

**Electronic supplementary material:**

The online version of this article (doi:10.1186/s12985-017-0687-7) contains supplementary material, which is available to authorized users.

## Background

New world alphaviruses such as Eastern equine encephalitis virus (EEEV) are the cause of highly pathogenic disease that can manifest as severe encephalitis in humans. Due to high infectivity, ability to induce devastating disease, ease of production, high degree of stability, and the potential for aerosolization, EEEV is considered a potential biological threat agent against both the Warfighter and civilians and is classified as a category B pathogen by the CDC and NIAID [1]. EEEV is a single-stranded, positive-sense RNA virus found in the eastern half of North America [2]. The disease is zoonotic in humans and severe infection can result in neurological invasion and encephalitis with mortality rates ranging from 33–70% [3, 4]. Encephalitic patients often experience severe symptoms of disease including high fever, headache, vomiting, general or focal seizures, focal weakness, cranial nerve palsies, and coma; long-term neurological sequelae may persist in survivors (~25%) and can include both motor and cognitive impairments [4–6]. EEEV is also pathogenic for equines and can produce mortality rates as high as 80–90% in horses [2]. Long-term neurological sequelae are observed in about 66% of surviving equines [2]. Until recently, EEEV was considered to consist of four genetic lineages [7]. Lineage I was considered to be the North American (NA) variant of EEEV, while lineages II, III and IV were the South American (SA) variants. The latter three lineages have now been classified as a new viral species, Madariaga virus [7]. EEEV strains (previously referred to as NA strains) are transmitted by the mosquito vector and are prevalent in coastal and swampy regions of the eastern United States; between 2003 through 2012, 89 cases of EEEV were confirmed in the US [3]. The geographical range of EEEV infections stretched from the Gulf to Atlantic coasts, with the majority of cases reported from Florida, Massachusetts, Alabama, North Carolina, New Hampshire, Louisiana, and Georgia [3].

The need for licensed vaccines and antiviral therapeutics for human use in the event of EEEV infection has fostered research efforts to characterize multiple animal models that can be used to assess efficacy of novel countermeasures, with respect to FDA guidance on the development of products under the Animal Rule [8]. Marmosets are a species of nonhuman primates (NHP) used in a wide range of research efforts for human disease such as reproductive biology, behavioral research, and biomedical research [9, 10]. Marmosets have previously been used to assess intranasal EEEV infection [11]. The small size of the marmoset allows for easy handling, reasonable housing space, and provides greater amounts of test material for research than traditional rodent models. The ease of breeding in captivity coupled with the fact that the numbers of animals in the wild are not threatened represent additional considerations for the justification and use of this species in biomedical research [11, 12]. However, in spite of previous use of the marmoset as a model of intranasal EEEV infection, there remains a need to understand how aerosol infection affects the host, as the aerosol route has been proposed to be the most likely route of infection used in a potential bioterrorism event. It is conceivable that aerosol exposure, may produce a different disease course, pathology, or disease outcome from the intranasal exposure [13, 14]. Infections generated by aerosol inhalation may produce different pathology and infection kinetics because aerosolized particles deposit deep within the alveolar spaces of the lung. Indeed, experimental evidence of EEEV infection in mice and human challenge models of Adenovirus infection demonstrate the variable effects on disease development and pathology that can be observed depending on the exposure route in both mice and humans [15, 16].

Cynomolgus macaques (cynos) have previously been used to model EEEV aerosol infection [17] using the EEEV FL91-4679 strain. In aerosol simulations, the cyno model has been shown to recapitulate many of the clinical signs of human infection, such as fever, seizures, and leukocytosis; however, other signs including increased liver enzymes and serum indicators of liver damage have not been observed in humans and suggest that while the cyno is a good model for some parameters, there is much that we do not understand about the pathology of the disease in different models. In addition to potentially disparate disease responses in the cyno in comparison to humans, the animals are becoming increasingly difficult and expensive to obtain for medical research [12, 18]. As an alternate nonhuman primate model, marmosets offer several distinct advantages over the traditional macaque model including the fact that marmosets do not harbor herpes B virus, breed well in captivity but are also abundant in the wild, and their small body size translates to decreased amounts of compound for drug testing or vaccine development [12].

The study design of the experiments described herein employed a staircase or up-and-down protocol to estimate the median lethal dose in marmosets wherein the marmosets were exposed to a range of aerosolized EEEV FL93-939 doses and followed for clinical disease and course of pathogenesis of EEEV disease. This assessment is critical for the development of the marmoset as an animal model that can potentially mimic human disease and to compare the responses in the marmoset to other animal models of EEEV disease following aerosol infection (e.g., cynomolgus macaques). We describe the differential effects of dose on the immune cell populations in the blood, blood chemistry, viral burden in tissue, fever, body weight, and changes in blood oxygen saturation levels as part of the clinical symptoms and pathology. These results, particularly the differential dose effects of aerosolized EEEV on fever, virus dissemination, neurological signs, and hematological parameters, reveal that the common marmoset is a promising model of lethal inhalational EEEV infection for use in the development of medical countermeasures.

## Methods

### Animals

Five healthy, adult common marmosets (*Callithrix jacchus*) (1 male, 4 females) were obtained from the United States Army Medical Research Institute of Infectious Diseases (USAMRIID) nonhuman primate colony and randomly assigned for exposure order. All marmosets were determined to be naïve to previous alphavirus infection and to be free of common opportunistic pathogens to include lymphocytic choriomeningitis virus (LCMV) *Salmonella*, *Shigella*, *Campylobacter*, and *Klebsiella pneumoniae*. All marmosets were sexually mature adults, ranged between 290 and 375 g in weight and were between 2 and 4 years of age at the time of study [18]. A mixture of male and female marmosets was used following FDA Animal Rule guidelines and the animals were at both the age and weight to be consistent with prime-age and size adults [8, 12, 18]. Using prime-aged, healthy adult animals was important to the characterization of the marmoset as a model of EEEV-induced encephalitis as these studies are ultimately intended to provide support of medical countermeasure development for the Warfighter. Marmosets were given water *ad libitum,* received the customary marmoset diet twice daily, daily dietary enrichment consisting of fresh fruit or yogurt, as well as conventional environmental enrichment including a mirror, nesting box, and perch bar. Animals were surgically implanted with subcutaneous Data Sciences International (DSI) TA-F40 telemetry implants to remotely monitor temperature. Approximately one week prior to aerosol exposure, animals were moved to biosafety level-3 (BSL-3) facilities at USAMRIID and housed in cages that were modified for marmosets. The animals were housed in rooms that were maintained at approximately 25 °C and on a 12 h light/dark cycle.

### Virus

The FL93-939 strain is a prototype North American EEEV strain. It was originally isolated from a pool of mosquitoes (*Culiseta melanura)* from Florida in 1993. The virus was a kind gift from Dr. Scott Weaver, University of Texas Medical Branch. The virus isolate history included one passage through C6/36 mosquito cells, one passage in suckling mouse brain, one passage in Vero cells (derived from African Green monkey kidney cells), and one passage in baby hamster kidney (BHK) cells at USAMRIID to produce the sucrose-purified stock. The passage history of the viral isolate may be important to understanding the virulence of the strain or potential adaptation for infection in humans or equines. Purified virus was diluted to the appropriate concentration in unsupplemented Eagle’s Modified Essential Medium with non-essential amino acids (EMEM w/NEAA) prior to aerosol exposure.

### Aerosol challenge

In preparation for aerosol challenge, marmosets were initially anesthetized with inhaled isoflurane and maintained with Ketamine-Acepromazine (Ket-Ace) during the aerosol exposure procedure. Each marmoset was exposed to aerosolized EEEV in a head-only exposure chamber contained in a class III biological safety cabinet inside a BSL-3 laboratory. Aerosol exposure was controlled and monitored using the Automated Bioaerosol Exposure system (ABES) [19]. Delivery of the target aerosol dose relied upon calculations of minute volume based on Guyton’s formula, taking into consideration: (1) the flow to volume ratio of the exposure chamber, (2) the starting EEEV concentration in the Collison nebulizer, and (3) the spray factor calculated from sham experiments using the virus stock [20]. All exposures were generated with a three-jet Collision nebulizer and air passing through the exposure chamber was collected for sampling in an all-glass impinger (AGI) [21]. Titer of the aerosolized agent collected in AGIs was determined for each exposure by viral plaque assay. The actual inhaled dose of EEEV was calculated based on the concentration and volume of the AGI samples, the estimated minute volume, and flow rate of the aerosol sampler using the following formula:

$$ \mathrm{Inhaled}\ \mathrm{Dose} = \mathrm{C}\left(\mathrm{AGI}\right)\ \mathrm{x}\ \mathrm{V}\left(\mathrm{AGI}\right)\ \mathrm{x}\ \mathrm{M}\mathrm{V}/\mathrm{Q}\left(\mathrm{AGI}\right) $$

Where inhaled dose (PFU) is calculated based on: C, the concentration (PFU/mL) of the virus sampled from the AGI; V, the volume contained in the AGI sample (mL); MV, the minute volume (mL/min) for each animal estimated from Guyton’s formula; and Q, the flow rate of the AGI sampler (mL/min).

### Telemetry analysis

Marmoset body temperature was recorded using the DataQuest A.R.T 4.1 system (DSI). The system was set to collect data every five minutes, beginning 7 days prior to aerosol exposure and continuing until day 28 post-exposure or earlier if study endpoint criteria were met. Statistical analysis was conducted as a Bayesian estimation of the distribution of daytime body temperature for each marmoset prior to aerosol challenge that was subsequently used to compute a credible range for body temperatures using SAS Markov chain Monte Carlo simulation procedures (PROC MCMC). Data analysis included temperature measurements that were compatible with life (i.e., ≤ 42 °C). The 99.7% credible range generated for each animal’s daytime body temperature was analogous to an interval of ±3 standard deviations (SD) for a normally distributed variable. All post-aerosol challenge temperature readings were compared to the expected temperature interval estimated for each animal. Temperature measurements above the upper limit of the estimated interval were noted as elevated and used to compute fever summary statistics.

### Observation, clinical evaluation, and study endpoint criteria

Marmoset clinical observations began three days prior to aerosol exposure for a baseline appearance and behavior appraisal and continued minimally twice daily post-exposure. Several factors were used when evaluating clinical signs of disease for each marmoset. Clinical observation parameters included: (1) neurological signs (0 = normal; 1 = loss of coordination, 2 = occasional tremors, 3 = loss of balance, 4 = frequent tremors/seizures); (2) temperature (0 = normal, 1 = > 1 °C above baseline, 2 = 2 °C above baseline, 3 = 3 °C above or below baseline, 15 = 4 °C below baseline); (3) appearance (0 = normal, 1 = reduced grooming, 2 = dull/rough coat or ocular nasal discharge, 3 = lethargy, 4 = piloerection or hunched up); (4) natural behavior (0 = normal, 1 = minor changes, 2 = little peer interaction, less mobile, 3 = no peer interaction, vocalization, or self-mutilation); and (5) provoked behavior (0 = normal, 1 = subdued when not stimulated, 2 = subdued when stimulated, 15 = unresponsive/weak, pre-comatose). To further assess the health status of the marmosets, animals were anesthetized every three days for collection of weight and to conduct a physical examination. During this time, blood samples were collected for complete blood count (CBC) analysis. White blood cell (WBC) counts were included in the clinical scoring of the animals. Scoring criteria for WBC were as follows: 0 = normal (0–10 K/μl); 1 = 10–12 K/μl; 2 = 12–14 K/μl; 3 = 14–20 K/μl; 4 = > 20 K/μl. In addition, blood oxygen saturation was determined every three days using a pulse oximeter. Study endpoint or euthanasia criteria took into consideration the clinical observation score, body temperature, and WBC results. Animals with a total clinical score ≥15 were considered to have reached a peak disease state and to have met the pre-determined criteria for study endpoint; moribund animals that reached this score were humanely euthanized.

### Plaque assay

Dissemination of infectious virus in blood and tissues was assessed by plaque assay. Briefly, USAMRIID Vero 76 cells, derived from African Green monkey kidney cells, were seeded on 6-well tissue culture plates and grown to 90–95% confluence. Samples were serially diluted in Hanks’ Balanced Saline Solution (HBSS). Cells were infected with 0.1 mL of serially diluted samples per well, in triplicate. Plates were incubated at 37 °C, 5% CO_2_ for 1 h with gentle rocking every 15 min. After 1 h, cells were overlaid with Eagle’s Basal Medium (BME) (Gibco A15950DK) containing 0.6% agar supplemented with 10% heat-inactivated FBS, 2% Penicillin/Streptomycin (10,000 IU/mL and 10,000 μg/mL, respectively), and further incubated for 24 h at 37 °C, 5% CO_2_. A second agarose overlay, prepared as described above, containing 5% neutral red vital stain (Gibco 02-0066DG) was added to wells and further incubated for 18–24 h for visualization of plaques and determination of viral concentration in each sample (virus plaque forming units [PFU] value).

### Semi-quantitative RT-PCR

Viral RNA was detected by reverse-transcriptase polymerase chain reaction (RT-PCR). Viral RNA from blood and swab samples was isolated using QIAamp Viral RNA mini kit (QIAGEN, Valencia, CA). RNA from tissue samples was isolated using RNeasy mini-protect kit (QIAGEN, Valencia, CA). Semi-quantitative RT-PCR was used for detection of viral RNA from oral swab samples as well as blood and tissue samples from the study marmosets. The EEEV viral RNA assay was designed to amplify a portion of nonstructural protein 1 (nsp1) of the Georgia 97 strain of EEEV using the following primers and probe: EEEV Forward 5′-TGCAAAgATGCTTTCC-3′, EEEV Reverse 5′-TCACCTGGTCTGTATCCA-3′, and a FAM (Carboxyfluorescein) and TAMRA (Carboxytetramethylrhodamine) dual-labeled TaqMan probe 5′-CAACGCAGGTCACTGACAAT-3′. Quantification of viral RNA in samples was achieved by comparison of unknown blood and tissue samples to an RNA standard generated from EEEV FL93-939 virus, that is the same virus strain used for the aerosol exposure of experimental animals. The standard curve ranged from 5.0 × 10^7^ (upper limit of detection [ULOQ]) to 5.0 × 10^2^ viral RNA copies (lower limit of detection [LLOQ]). Repeated attempts to amplify virus below the LLOQ failed to consistently and reliably demonstrate amplification, thus the LLOQ was set at 5.0 × 10^2^ viral RNA copies. Positive and negative extraction controls were created by supplementing uninfected NHP blood with a known amount of EEEV FL93-939 virus (5.0 × 10^4^ viral genomic copies) and RNase-free water, respectively.

### Pathology

Necropsies were performed on each marmoset under BSL-3 conditions. Tissues were collected from all major organs in the body for histopathological and immunohistochemical assessment. Tissues were immersion fixed in 10% buffered formalin and held in biocontainment for a minimum of 21 days. Tissues for histopathology underwent routine histologic processing, were embedded in paraffin, sectioned, and stained with hematoxylin and eosin. Immunohistochemistry was performed on all tissue sections using a rabbit anti-Alphavirus (#1140) antibody (1: 8000 dilution) and a commercial immunoperoxidase detection kit (EnVision System, Dako Corp., Carpinteria, CA). After pretreatment with a TRIS/EDTA buffer (pH 9.0) at 97 °C for 30 min, primary and secondary antibodies were applied and the slides were incubated with substrate-chromagen solution according to the manufacturer’s recommendations. Sections were counterstained with hematoxylin.

## Results

### Aerosol exposure

To evaluate the common marmoset (*Callithrix jacchus*) as an animal model of aerosolized EEEV infection, five marmosets were challenged with increasing doses of EEEV in a head-only aerosol exposure chamber and then observed to determine how well the marmoset model emulated human disease. The individual inhaled doses obtained for each of the exposed marmosets were: 2.40 × 10^1^ PFU, 1.15 × 10^3^ PFU, 1.20 × 10^4^ PFU, 9.76 × 10^4^ PFU and 7.95 × 10^5^ PFU. The escalating doses of aerosolized EEEV elicited various responses in the animals tested; one marmoset survived, three marmosets became moribund and reached predefined study endpoint/euthanasia criteria (defined as a cumulative score of ≥ 15 taking into consideration the clinical observation score, body temperature, and WBC of each animal), and one marmoset succumbed to disease. In addition to survival, clinical and physiological changes were assessed and diagnostic tests were used to monitor blood and tissue dissemination of the virus in the marmosets.

### Clinical signs

**Fig. 1:**
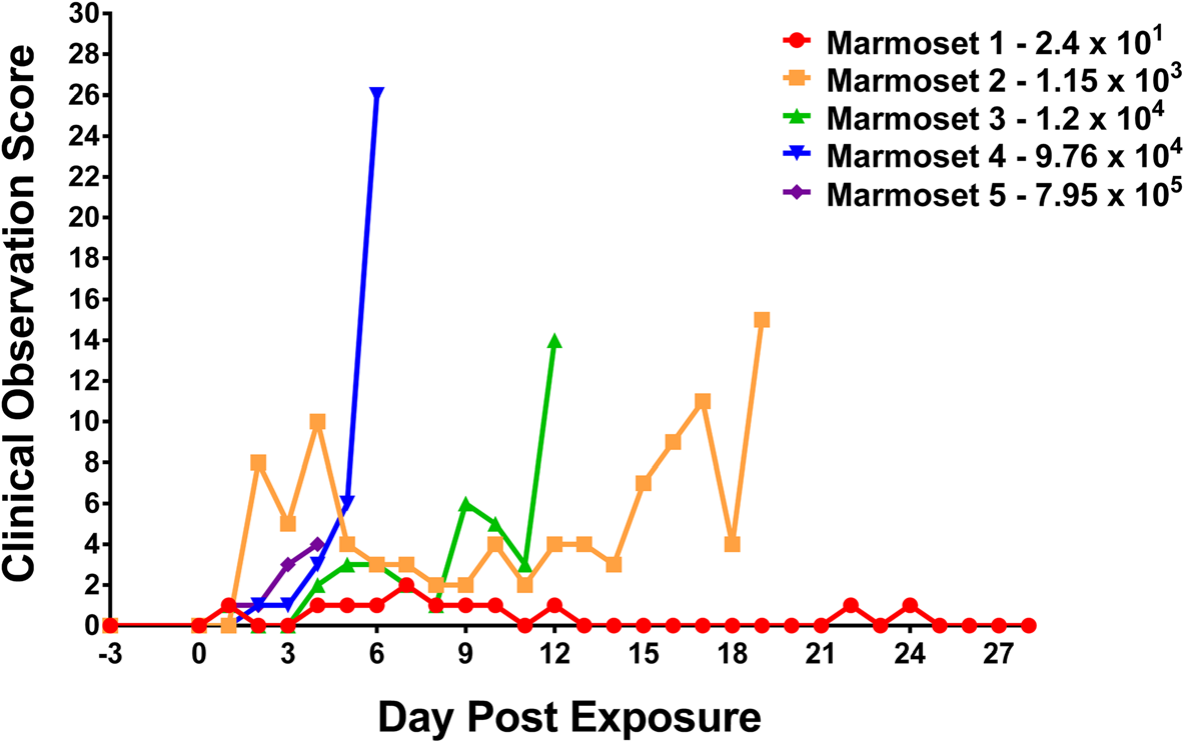
Clinical observation scores in marmosets with increasing doses of aerosolized EEEV. Clinical observation parameters included: (1) neurological signs, (2) temperature, (3) appearance, (4) natural behavior, and (5) provoked behavior. Animal behavior was noted and the sum of the score for each parameter was calculated. The values correspond to the highest score obtained for a marmoset per day

Marmosets were observed for the development of clinical signs indicative of EEEV disease in accordance with the scoring system described in the Material and Methods section. Briefly, clinical observation parameters included neurological signs (e.g., loss of coordination, tremors, or seizures), fever or hypothermia, appearance (e.g., changes in coat/grooming, lethargy, hunched posture), natural behavior (e.g., reduced interaction with peers or observers, vocalizations, reduced activity), and provoked behavior (e.g., changes in response to stimulation or pre-comatose posture). Subtle changes in the appearance and behavior of animals were observed as early as day 2 following EEEV aerosol exposure, and by day 3, most marmosets had presented with clinical signs, with the exception of marmoset #1 (2.4 × 10^1^ PFU) (Fig. 1). Marmoset #1 (inhaled dose: 2.4 × 10^1^ PFU) survived and displayed minimal observable clinical changes, mainly between days 4 and 12. Ruffled fur and reduced grooming of marmoset #1 were the first visual signs of infection to be noted in the daily observations. Survival time following aerosol exposure was observed to be inversely proportional to the inhaled dose. Marmoset #2 (1.15 × 10^3^ PFU) received an inhaled dose that resulted in a prolonged study observation period (marmoset reached euthanasia criteria on day 19 post-exposure). Marmoset #3 received an intermediate dose of 1.2 × 10^4^ PFU that corresponded to a survival profile observed to fall between the lower and higher doses; the marmoset reached euthanasia criteria on day 12 post-exposure. Marmoset #4 (9.76 × 10^4^ PFU), receiving an aerosol dose approximately one half-log less than marmoset #5, displayed a similar, but more prolonged course of disease than that of marmoset #5. The clinical scores for marmoset #4 were higher between days 3 and 4 and the animal reached euthanasia criteria by day 6, two days later than marmoset #5. Marmoset #5, receiving the highest dose of aerosolized EEEV (7.95 × 10^5^ PFU) showed clinical signs as early as day 2 following exposure and succumbed to the disease on day 4, displaying an abbreviated disease course due to the severity of infection. Lethargy, decreased interaction, slight piloerection, tremors, and lack of balance were noted in several of the animals that became moribund and met euthanasia criteria; these marmosets received higher inhaled doses of EEEV. At later times in the disease course, marmosets became more subdued in their behavior and response to stimulation was absent, even when provoked.

**Fig. 2:**
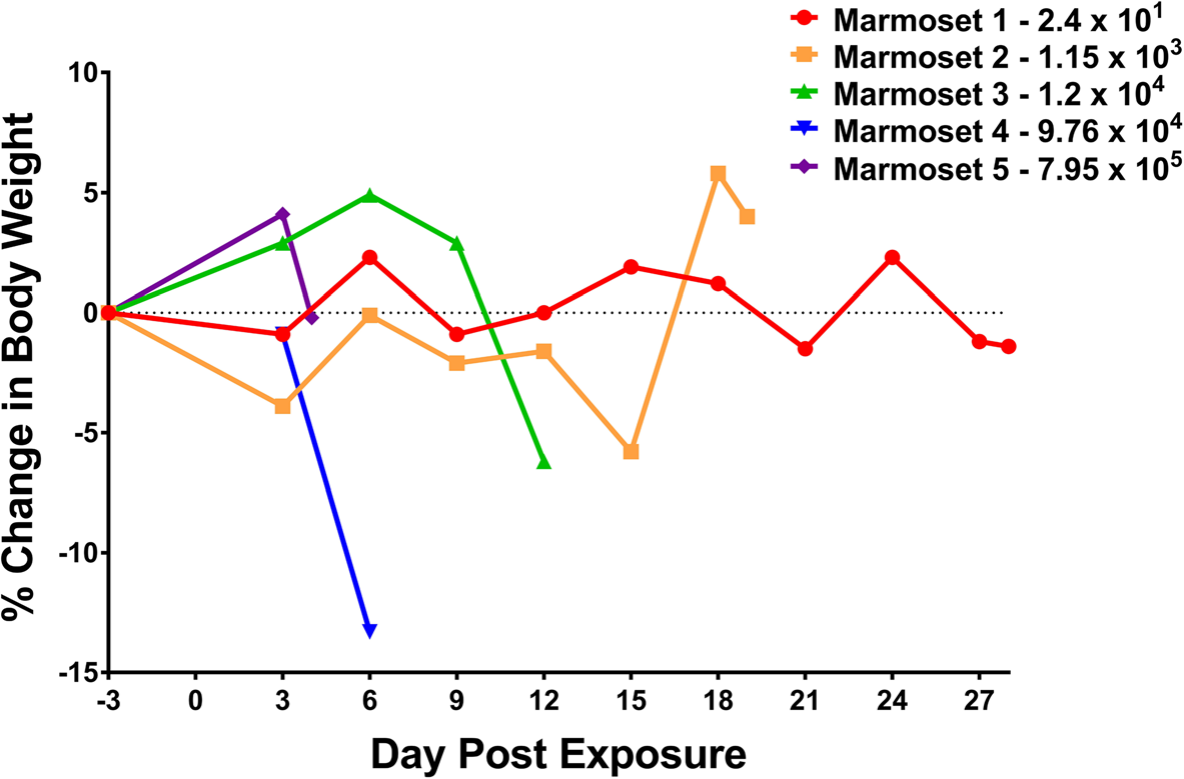
Effect of aerosolized EEEV on total body weight of marmosets. Marmoset weights were recorded every third day during anesthetized physical observation. Weight loss was most evident in marmosets #3 and #4. The disease course, including weight loss, for marmoset #2 followed a more prolonged course, consistent with the lower inhaled dose of EEEV that the animal received. Marmoset #5 failed to show a meaningful decrease in body weight since this animal succumbed to disease on day 4 post-exposure

The effect of EEEV exposure on marmoset body weight was assessed. Considerable weight loss was observed between day −3 and study endpoint in marmoset #3 (1.2 × 10^4^ PFU) and marmoset #4 (9.76 × 10^4^ PFU) with loss in body weight of 6.25 and 13.3%, respectively (Fig. 2). The rapid progression of disease into lethality observed for marmoset #5, having received the highest EEEV dose, resulted in a marginal decrease in weight between day −3 and day 4 post-exposure (endpoint). For doses below 1.2 × 10^4^ PFU, a 4.02% weight increase was observed for marmoset #2 and a modest 1.34% decrease for marmoset #1. These results indicate that weight loss was observed to be greater in marmosets that received higher aerosol doses. In fact, the dose range encompassing marmosets #3 and #4 was the critical interface where clinical manifestations and body weight loss were most evident; these results corroborate changes in weight that have been reported for other species such as mice, hamsters, and guinea pigs [15, 22, Erwin-Cohen, unpublished data].

**Fig. 3:**
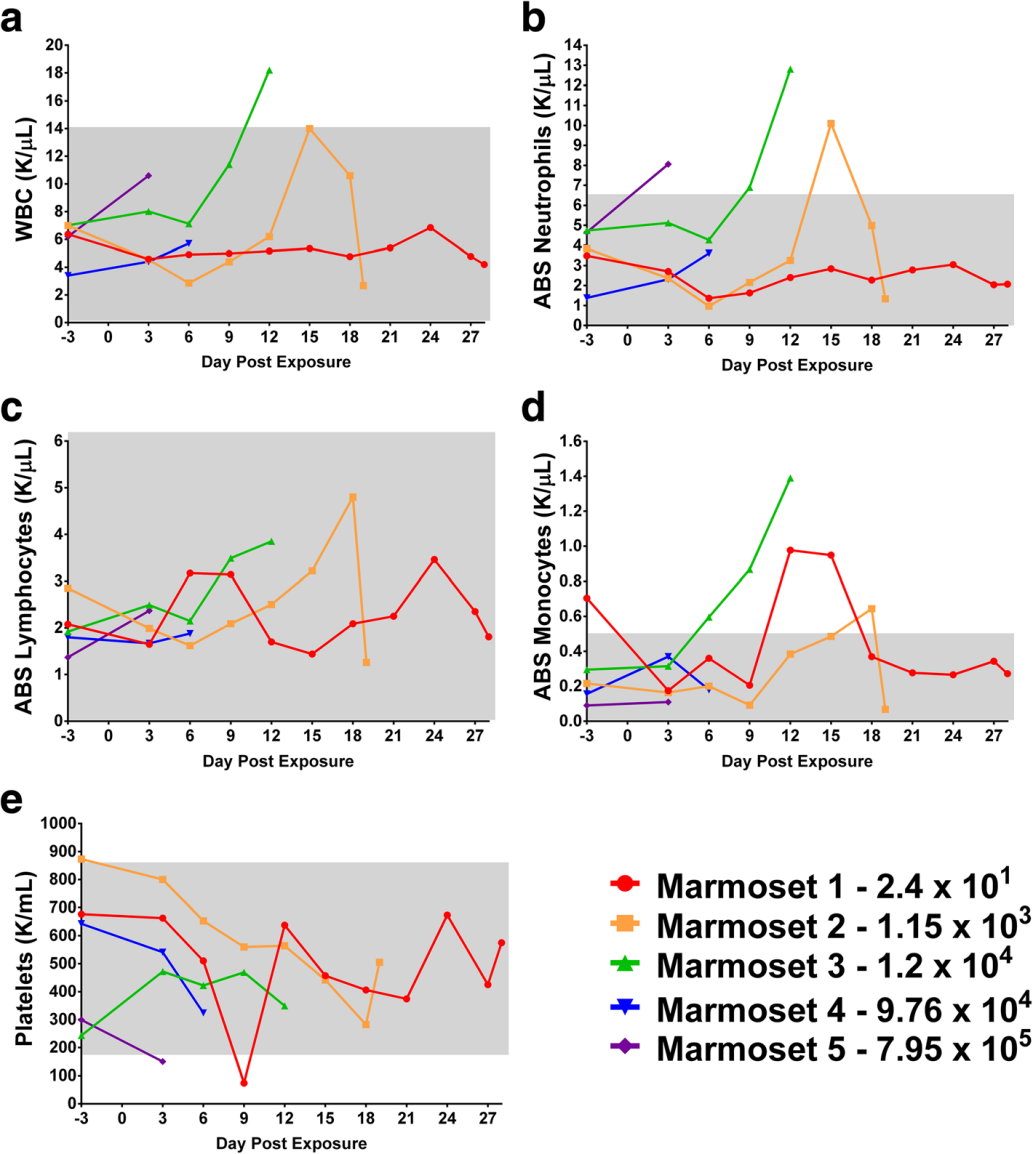
Hematological changes in marmosets after aerosolized EEEV challenge. The doses of EEEV to that the marmosets were exposed are indicated in the bottom right of the figure. Grey areas over the graphs correspond to normal value ranges for marmosets. The graphs show the results for (**a**) white blood cell counts, (**b**) neutrophils, (**c**) lymphocytes, (**d**) monocytes, and (**e**) platelets in the infected marmosets through time

The hematological changes occurring in marmosets receiving distinct aerosol doses were next addressed. For this purpose, CBCs were performed on samples collected pre-exposure and then on days 3, 6, 9, 12, 15, 18, 21, 24, 27 and at study endpoint (Fig. 3). Marmosets that received lower doses of EEEV initially displayed a decrease in WBCs on days 3 to 9 post-exposure (Fig. 3a). Overall, an increase in total WBC count was observed in marmosets that progressed through the disease course, consistent with reports of leukocytosis in human cases of EEEV infection (Fig. 3a). Sharp increases in WBCs and neutrophils (a clear contributor to the overall increase in WBCs) occurred during the peak days of clinical disease (Fig. 3a and b, respectively) for marmosets that received a lethal dose. It was noted that marmoset #2 also displayed acute increases in WBCs and neutrophils on day 15, but the levels of both returned to pre-exposure levels by days 15–18 as the animal became moribund. The increase in the number of lymphocytes (Fig. 3c) followed a trend similar to that of the neutrophils (Fig. 3b) for the marmosets receiving lethal aerosol doses. A biphasic lymphocyte response was noted for the surviving marmoset #1. A steady increase in monocytes was noted in marmosets #2 and #3 over the course of infection, though there was a sharp increase noted for marmoset #2. The increase in monocytes observed for marmoset #2 was most intense between days 9–18. Likewise, monocytosis was observed for marmoset #1 (the lone surviving animal) between days 9 and 18, but resolved thereafter until the end of study (Fig. 3d). In general, the number of platelets showed a downward trend over the course of infection. The surviving marmoset #1 had a substantial drop in platelets between days 3 and 9; however, this was resolved by the end of the study (Fig. 3e). Mild thrombocytopenia has been observed in cynos experimentally infected with a human isolate of EEEV (strain V105-00210) [Burke, personal communication]; however, this is the first observation of thrombocytopenia in marmosets in response to EEEV infection. No animal displayed signs of anemia during the course of study; levels for hemoglobin, hematocrit, and red blood cells (RBC) remained within normal levels throughout the study (data not shown). The leukocytosis observed in marmosets infected with EEEV agrees with published reports of similar effects of EEEV infection in humans [6, 7]. The normal hematological and serum chemistry ranges in marmosets were established from the USAMRIID colony of marmosets prior to the study (Additional file 1: Table S1).

**Fig. 4:**
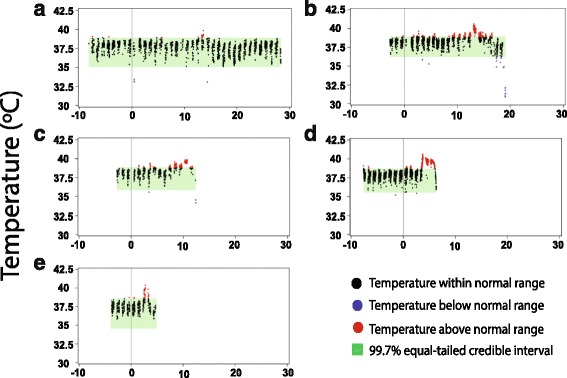
Fever response in marmosets after EEEV aerosol challenge. Variation in body temperature is shown for (**a**) marmoset #1, (**b**) marmoset #2, (**c**) marmoset #3, (**d**) marmoset #4 and (**e**) marmoset #5. Fever was determined by comparing baseline body temperatures of the marmosets with temperatures measured after aerosol exposure. Baseline temperatures were collected every five minutes from as early as 7 days before challenge. Telemetry collection continued after exposure until study endpoint (up to 28 days post-exposure). Average daily elevations in body temperature and any residual temperature data above 3 SD were used to compute fever duration

Fever onset and duration followed a dose-dependent pattern (Fig. 4). With the exception of marmoset #1 that received the lowest dose, all other marmosets developed fever as defined by a temperature change that was 3 SD over the average body temperature for that animal and occurring on at least three consecutive readings (Fig. 4 and Table 1). Although marmoset #1 revealed sporadic spikes in temperature resembling fever, those spikes did not occur continuously (data not shown). This animal was the only one that survived aerosol exposure until the planned duration of the study. In marmosets receiving the highest EEEV doses (marmosets #4 and #5), fever occurred earlier and had a shorter duration (see Fig. 4d and e, and Table 1), and for lower-inhaled doses, fever was more prolonged as was the course of the disease (see Fig. 4b and c, and Table 1). The common aspect for the marmosets that met euthanasia criteria was an increase in body temperature (fever), at times considerable, immediately followed by a noticeable drop in the animal’s body temperature as the animal became moribund (Fig. 4b-e). In contrast, the surviving marmoset (marmoset #1) retained a steady body temperature throughout the course of the study without onset of fever. In the exposed marmosets, fever persisted for 25 h to 122 h. Marmoset #2, which received the lowest lethal aerosol dose of EEEV, exhibited the most prolonged period of fever (Table 1). In all animals, fever peaked at approximately 40 °C, except for marmoset #5 that peaked at 43.6 °C.

**Table 1:** Summary of febrile response in marmosets exposed to increasing doses of aerosolized

Animal	Inhaled Dose	Fever onset	Duration	Fever Peak Temp	Last Temp Taken	Time of Death
ID	(PFU)	(day)	(hr)	(°C)	(°C)	(day post exposure)
1	2.40 × 10^1^	N/A	0	N/A	36.0	28
2	1.15 × 10^3^	10	122	40.1	31.4	19
3	1.2 × 10^4^	8	64	39.8	34.2	12
4	9.76 × 10^4^	3	53	40.6	36.1	6
5	7.95 × 10^5^	2	25	43.6	37.5	4

**Fig. 5:**
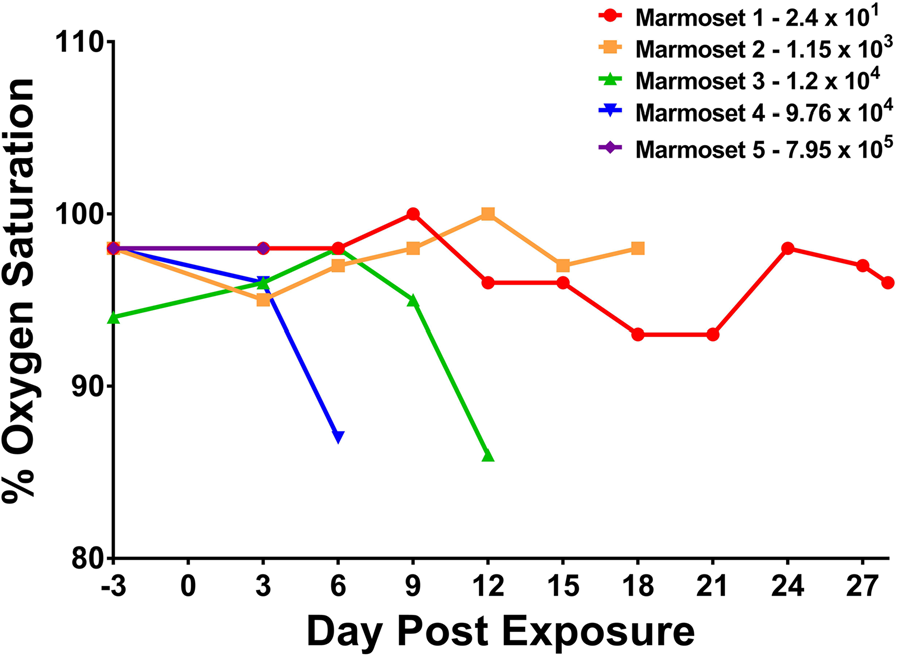
Changes in blood oxygen saturation in the marmosets was measured prior to and following exposure to aerosolized EEEV. Blood oxygen saturation values were determined using a pulse oximeter every 3 days while animals were under anesthesia for physical examination

Oxygen saturation levels in blood were also collected to further evaluate the health status of the marmosets in the study. These data were obtained using a pulse oximeter (Fig. 5). Variation in oxygen saturation was more evident in marmosets receiving intermediate doses of EEEV aerosol (i.e., marmoset #3: 1.20 × 10^4^ PFU and marmoset #4: 9.76 × 10^4^ PFU) in Fig. 5. The sharp change in oxygen saturation observed for the marmosets exposed to intermediate doses were not noted in those animals receiving either the highest or the two lowest EEEV doses. No dramatic changes in percent oxygen saturation were noted in marmosets that received highest and two lower doses of EEEV. Compared to pre-exposure values (day −3), percent oxygen saturation decreased from 94% at day −3 pre-aerosol exposure to 86% at study endpoint (day 12) for marmoset #3 (Fig. 5). A similar drop was seen for marmoset #4 (9.76 × 10^4^ PFU) from 98% (day −3) to 87% (study endpoint, day 6).

### Virus dissemination

The presence of virus in tissues, whole blood, and oral swabs collected from infected marmosets was assessed either by plaque assay and RT-PCR. Tissues were collected from the marmosets at necropsy, while the blood and oral swab samples were serially collected prior to exposure and every 3 days following aerosol exposure at physical examination while the marmosets were under anesthesia. Infectious virus was found to be widely disseminated in tissues of marmosets that received the higher EEEV aerosol doses (marmosets #3 through #5) (Table 2). Marmoset #1 received a non-lethal dose and was the only animal with no detectable EEEV in tissues by either plaque assay or RT-PCR (Table 2). Virus was detected in brain by at least one of the detection methods in all marmosets that received a lethal dose (marmosets #2 through #5) (Table 2). The liver and kidney had detectable levels (by at least one method) of virus in the marmosets that received the three highest EEEV doses (marmosets #3 through #5). EEEV was detected in the mesenteric lymph node and heart of marmosets receiving higher doses of aerosolized EEEV (Table 2). Infectious virus was detected in the lungs by plaque assay only in the marmoset that received the highest inhaled dose of EEEV, suggesting that the virus is rapidly cleared from the lungs during inhalational infection (Table 2). EEEV was detected in the inguinal and mandibular lymph nodes by both plaque assay and RT-PCR in marmoset #3, an animal that received an intermediate dose of EEEV and survived to day 12 post-exposure. The adrenal gland was positive by both plaque assay and RT-PCR in the marmoset that received the highest dose.

**Table 2:** Viral Plaque Assay and RT-PCR results in solid tissues from marmosets exposed to aerosolized EEEV

	Animal ID (Inhaled Dose, PFU)									
			marmoset #2 (1.15 × 10^3^)		marmoset #3 (1.2 × 10^4^)		marmoset #4 (9.76 × 10^4^)		marmoset #5 (7.95 × 10^5^)	
	Plaque Assay (PFU/g)	PCR	Plaque Assay (PFU/g)	PCR	Plaque Assay (PFU/g)	PCR	Plaque Assay (PFU/g)	PCR	Plaque Assay (PFU/g)	PCR
Salivary Gland	-	-	-	-	1.4E + 04	-	-	-	-	-
Adrenal Gland	-	-	-	-	-	-	-	-	1.2E + 04	+
Pancreas	-	-	-	-	-	-	-	-	-	-
Lung	-	-	-	-	-	-	-	-	2.0E + 04	-
Spleen	-	-	-	-	-	+	-	+	-	-
Axillary LN	-	-	-	-	-	-	-	+	-	-
Kidney	-	-	-	-	-	+	1.3E + 04	+	9.5E + 03	-
Brain	-	-	-	+	1.6E + 07	+	4.2E + 04	+	-	+
Heart	-	-	-	-	-	-	3.6E + 03	-	-	+
Liver	-	-	-	-	-	+	1.9E + 04	-	-	+
Inguinal LN	-	-	-	-	4.2E + 04	-	-	-	-	-
Mandibular LN	-	-	-	-	4.2E + 04	+	-	-	7.4E + 03	-
Trachbronical LN	NT	NT	-	-	-	+	5.0E + 04	-	-	+
Mesentric LN	-	-	-	-	-	-	7.3E + 04	-	-	+
Popliteal LN	-	-	-	-	-	+	-	-	-	-

*NT* no tissue

+ = positive assay result; − = negative assay result

When blood samples were assessed by plaque assay, viremia was not detected in any animals at any time point (data not shown). Only marmoset #1 tested positive for EEEV RNA in the blood by RT-PCR at days 9 and 12 post (Table 3). Oral swabs were also negative for EEEV by either plaque assay or RT-PCR (data not shown). Comparison of results between plaque assay and RT-PCR should be approached with caution as differences in sensitivity and sample recovery between these two assays may help explain why some samples are positive for one assay but not the other.

**Table 3:** Detection of EEEV in blood from infected marmosets by RT-PCR

Animal ID (Inhaled Dose, PFU)	Sample Collection Day												
	−3	3	4	6	9	12	15	17	18	19	21	24	27
1 (2.40 × 10^1^)	-	-	-	-	+	+	-	-	-	-	-	-	-
2 (1.15 × 10^3^)	-	-	ND	-	-	-	-	-	-	-			
3 (1.20 × 10^4^)	-	-	ND	-	-	-							
4 (9.76 × 10^4^)	-	-	ND	-									
5 (7.95 × 10^5^)	-	-	ND										

*ND* Not done

+ = positive PCR result; − = negative PCR result

### Pathology

**Fig. 6:**
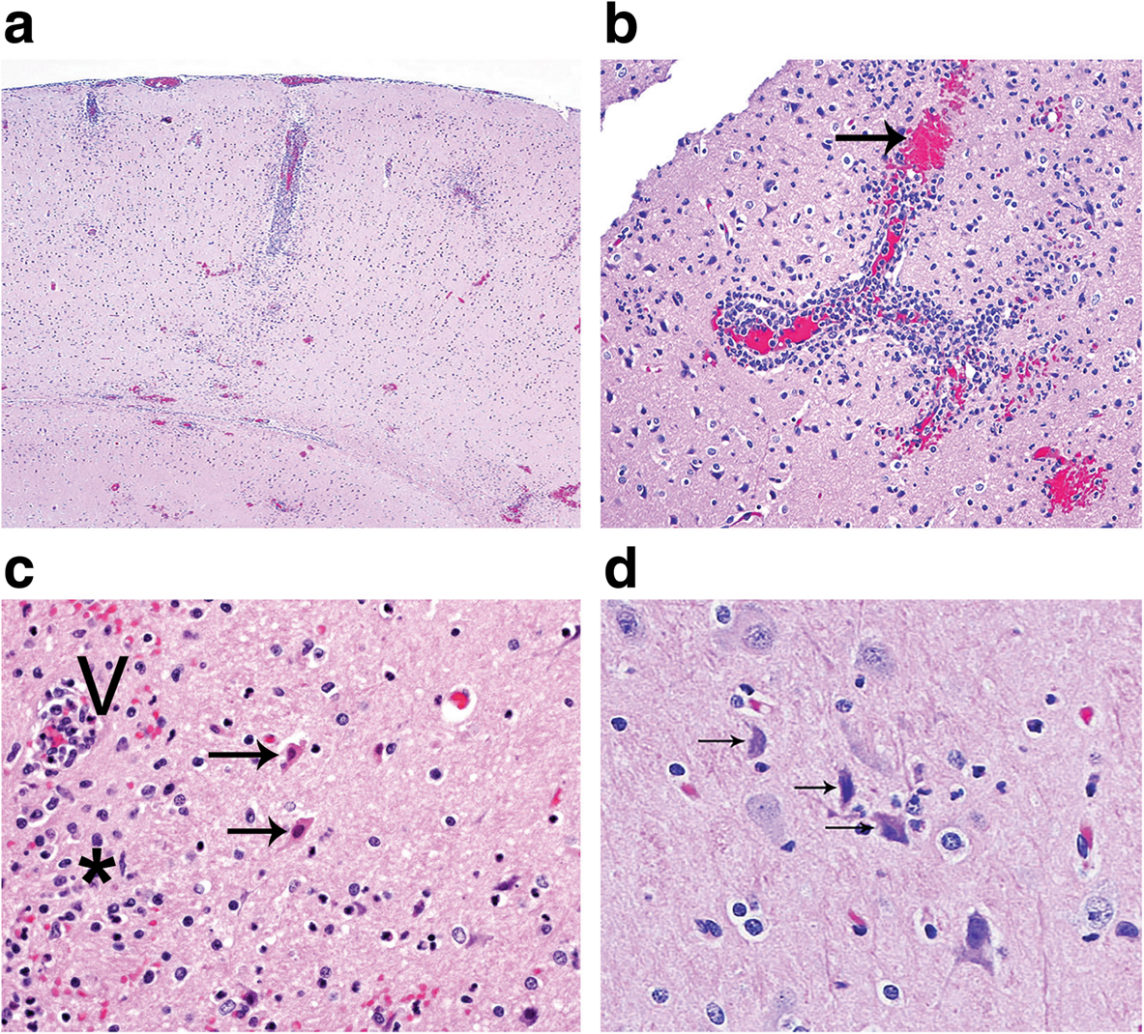
Histopathology following exposure to aerosolized EEEV. **a** Examination of the frontal cortex of the brain revealed the presence of multifocal meningoencephalitis with hemorrhage (HE; magnification, 4×). **b** Blood vessel in frontal cortex displayed vasculitis with perivascular hemorrhage (*arrow*) (HE; magnification, 20×). **c** In the corpus striatum, two neurons (*arrows*) showed hypereosinophilic perikaryon (cytoplasm) suggesting necrosis. Vasculitis (V) with perivascular hemorrhage was seen in an adjacent vessel and gliosis (*asterisk*) in the surrounding neuropil (HE; magnification, 40×). **d** In the pons, three centrally located neurons (*arrows*) were observed that were shrunken and angular with hypereosinophilic perikaryon and deeply basophilic (hyperchromatic) nuclei (HE; magnification, 60×). Images are from marmoset #4 exposed to 9.76 × 10^4^ PFU of EEEV

No gross lesions were present at necropsy in any animal, irrespective of dose or disease outcome. Histologic evaluation revealed few changes between doses in the animals that reached euthanasia criteria. Clinical disease progressed similarly, but the observations varied temporally depending on the aerosol dose with the exception of the observation of vasculitis. Vasculitis was apparent in marmosets that received doses greater than 1.0 × 10^4^ PFU. The histologic changes included acute to subacute meningoencephalitis (Fig. 6a) with neuronal necrosis and prominent vasculitis (Fig. 6b) present within the brain of marmosets infected at higher doses (>1.0 × 10^4^ PFU). The surviving marmoset did not have any histologic changes present. Of those with histologic lesions, the portions of the brain most severely affected were the frontal cortex, corpus striatum, thalamus; mesencephalon, pons, medulla oblongata, and cerebellum. The meningoencephalitis consisted of infiltration of mononuclear inflammatory cells with high numbers of neutrophils. Additional changes described included neuronal cell death (Fig. 6c and d), gliosis, satellitosis, edema, and vasculitis. Hemorrhage was occasionally present. Table 4 summarizes the pathological findings observed in the EEEV-infected marmosets.

**Table 4:** Observed Pathologies in marmosets challenged with EEEV by the aerosol route

Marmoset	Inhaled Dose (PFU)	Time to Death Postexposure	Brain		Retina	
			Meningoencephalitis	Vasculitis	Retinitis	Vasculitis
1	2.40 × 10^1^	28 ^a^	-	-	-	-
2	1.15 × 10^3^	19	++	-	-	-
3	1.2 × 10^4^	12	+++	+++	-	-
4	9.76 × 10^4^	6	++	++	-	-
5	7.95 × 10^5^	4 ^b^	+++	+++	-	-

^a^ = Survivor, ^b^ = Succumbed to disease

+ = mildly present, ++ = moderately present, +++ = strongly/markedly present

**Fig. 7:**
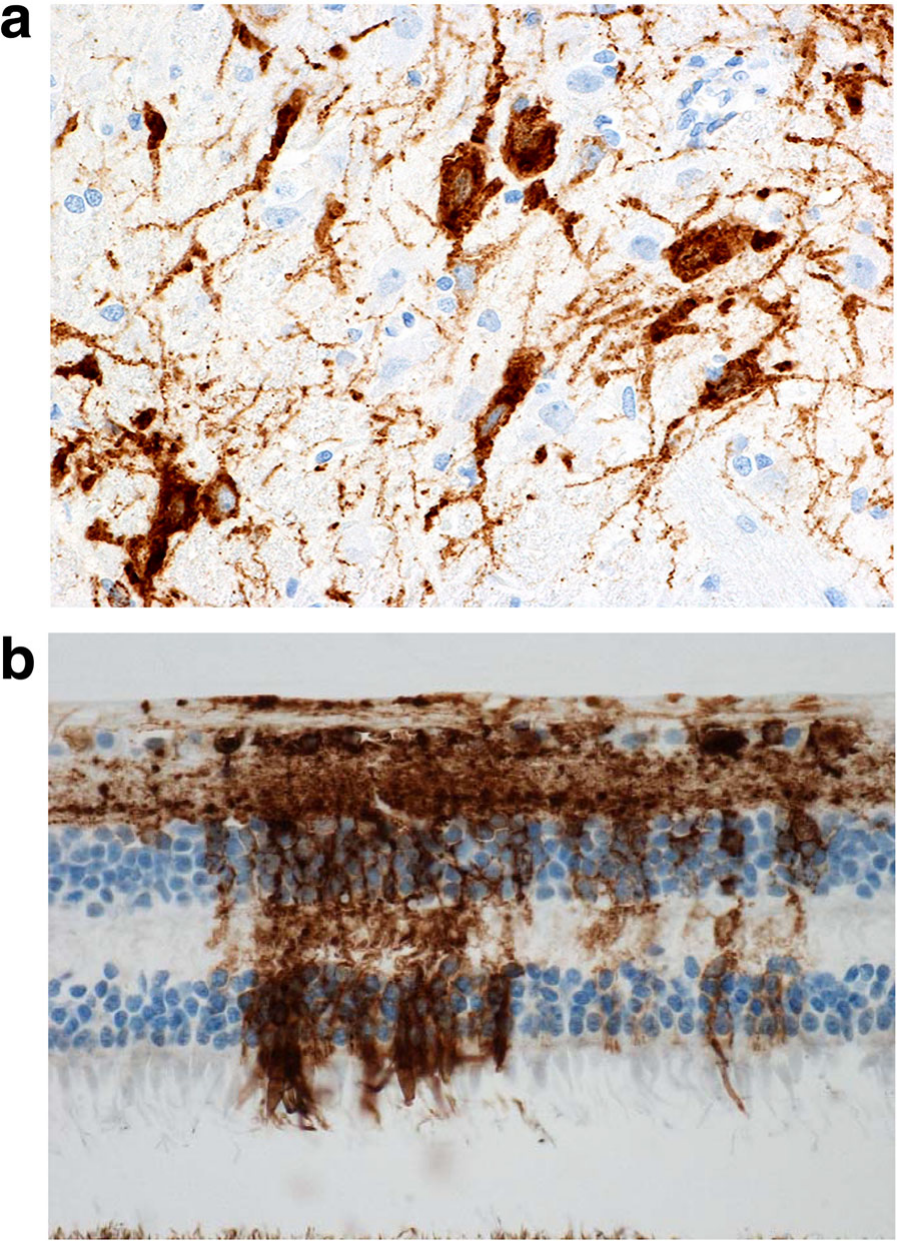
Immunohistochemistry analysis for marmosets exposed to aerosolized EEEV. The image in Panel **a** from the pons reveals widespread positive antigen staining of neurons in the brain, indicating the presence of EEEV viral RNA(immunohistochemistry; magnification, 40×). Panel **b**, immunohistochemical staining for the presence of antigen in the retina of marmoset #4. Depicted from the top of the image toward the bottom are: inner limiting membrane, optic fiber layer, ganglion cell layer, inner plexiform layer, inner nuclear layer, outer plexiform layer, outer nuclear layer, and external limiting membrane. The photo receptor layer appears predominantly negative (bottom of image) and the pigment epithelium is barely present in the image. Both images are from marmoset #4 exposed to 9.76 × 104 PFU of EEEV

Presence of EEEV in select tissues was demonstrated using immunohistochemistry (IHC). Positive IHC staining for EEEV antigen was observed in neurons within the brain (Fig. 7a) and the retina (Fig. 7b). Regions of brain with strongest IHC labeling were the frontal cortex, corpus striatum, thalamus, mesencephalon, and pons. Other tissues with positive IHC staining included macrophages/dendritic cells within the mandibular and axillary lymph nodes, interstitial cells of the ovary, and cells of the inner ear (data not shown). Nasal turbinates, nasal septum, and tooth pulp were also examined but were negative for the presence of viral antigen by IHC staining. No histologic changes were noted in the retina, despite positive IHC antigen staining (Tables 4 and 5).

**Table 5:** EEEV Immunohistochemistry Results for Marmosets challenged with EEEV by the aerosol route

Marmoset	Inhaled Dose (PFU)	Time to Death Postexposure	Brain	Retina
1	2.40 × 10^1^	28 ^a^	-	-
2	1.15 × 10^3^	19	+	-
3	1.2 × 10^4^	12	+++	+
4	9.76 × 10^4^	6	+++	+++
5	7.95 × 10^5^	4 ^b^	+++	-

^a^ = Survivor, ^b^ = Succumbed to disease

+ = mildly present, ++ = moderately present, +++ = strongly/markedly present

## Discussion

New World alphaviruses represent a recognized biological threat that can be intentionally delivered by aerosol; new vaccines and therapeutics that are being developed must be tested for efficacy against alphavirus inhalation exposure [23]. The purpose of the present work was to investigate the common marmoset (*Callithrix jacchus*) as a model of inhalational EEEV disease. There is an acknowledged need within the defense research community to characterize a variety of animal models of alphavirus infection with respect to FDA requirements under the Animal Rule for licensure of vaccines or therapeutics for pathogens for which human efficacy trials are not feasible or ethical. Furthermore, there is a need to understand the pathology of multiple serotypes of alphaviruses, particularly VEEV and EEEV, in multiple animal models that recapitulate human disease as closely as possible. [8, 13, 14]. Several animal species have previously been examined as models of EEEV infection including mice, hamsters, guinea pigs, and macaques; however, no single model presents an ideal candidate to recapitulate human disease for medical countermeasure testing.

Mice and hamsters are highly sensitive inhalation models of alphavirus disease, yet the vascular manifestation that is usually fatal in humans represents a shortfall in the murine model as mice do not develop perivascular infiltrates and only minimal encephalitis in the brain, while the fulminant course of disease and rapid time to death is a limitation in the utility of the hamster ([15, 24] Erwin-Cohen, personal communication]). South American strains of EEEV appear to produce a less virulent form of disease in humans than do North American strains [25]; experiments to characterize the guinea pig model of EEEV infection (with both North and South American strains) have demonstrated some characteristics that are consistent with human disease, such as fever, lethargy, and pathology of neuroinvasion [22]. Guinea pigs also demonstrate some inconsistent characteristics that limit the utility of the model; for example, aerosol infection in guinea pigs produces similar lethal susceptibility to either South American or North American strains of EEEV [22], in contrast to what has been observed for human EEEV infection. These results indicate species-specific differences in host-pathogen interactions. The marmoset was demonstrated to respond to intranasal infection with South American and North American strains in a manner that is similar to human disease with regard to differential susceptibility and thus warranted further investigation of this species as model of inhalational EEEV infection [11].

The effect of inhalational EEEV infection has been assessed in both rhesus and cynomolgus macaque nonhuman primate species and in equine models [17, 26]. Nathanson and colleagues infected juvenile rhesus macaques with a North American strain of EEEV via the intranasal route and produced fatal encephalitis [26]. However, further characterization of the rhesus model with adult primates or using the aerosol route of infection has never been addressed. In their characterization of the cynomolgus macaque as a model of aerosolized EEEV, Reed et al. [17] described the clinical disease course in cynos that had been exposed to two high doses of aerosolized North American EEEV strain FL91-4679 that differed by only log^0.5^. Reed observed signs consistent with human infection including fever, increased heart rate, and leukocytosis, but also noted additional features of the EEEV disease in cynos that were inconsistent with human infection including elevated liver enzymes and serum markers of liver damage [17].

The report on the common marmoset comparing virulence of intranasal infections between North American (NA) and South American (SA) strains of EEEV (re-classified as Madariaga virus) was promising as comparable responses to humans were found in the marmoset model [11]. The fact that in this study marmosets developed vasculitis and have previously been reported to produce neutralizing antibodies following infections with low virulence alphavirus strains was interpreted as a promising indication of this model as being suited for inhalational studies. Collectively, these factors prompted us to evaluate the marmoset for its suitability as a model of inhalational EEEV disease.

To determine the effect of the aerosol dose on the infectivity and disease development and estimate the medial lethal aerosolized dose of EEEV for the marmoset, inhaled doses ranging from 2.4 × 10^1^ PFU to 7.95 × 10^5^ PFU were tested. Markers of EEEV infection and disease were observed. As the dose increased, so too did the severity of the clinical disease course; marmosets reached euthanasia criteria progressively earlier and displayed a more abbreviated list of clinical signs and symptoms, revealing the dose-dependent nature of EEE disease manifestation. A gradient of disease severity was observed within the lethal doses. Marmosets receiving the two highest doses of EEEV succumbed to disease by days 4 and 6, respectively. This dose range revealed a similar effect as that observed when marmosets were exposed by the intranasal route with 1.0 × 10^6^ PFU of the EEEV FL93-939 strain [11]. In Adams’ work, the time of death and the host responses observed were similar to what we observed in the present study [11]. However, in our study, marmosets exposed to lower doses of EEEV displayed a protracted time to death, and the surviving marmoset exposed to the lowest aerosol dose remained nearly asymptomatic throughout the course of the study. The responses of the surviving marmoset more closely resembled those of marmosets exposed intranasally to the SA strain of EEEV (Madariaga virus) in Adams’ work; suggesting a dose threshold for NA strain toxicity that responds similarly to high intranasal doses of the less virulent SA strain (Madariaga virus) [11]. From the survival and clinical disease results that were observed in the present study, the median lethal aerosol dose in marmosets was estimated to be 2.05 × 10^2^ PFU.

Loss of body weight was demonstrated to be a factor in the disease pathology of intranasal EEEV infection in marmosets [11]. Loss of body weight has also been demonstrated in rodent models of EEEV infection but has never been demonstrated to be altered significantly in larger animal species such as macaques or humans [6, 15, 17]. We observed a loss of body weight between day −3 and study endpoint in response to aerosolized EEEV for marmosets #3 and #4 (1.2 × 10^4^ PFU and 9.76 × 10^4^ PFU, respectively). Infection with doses below this range either had no effect on body weight or the marmoset gained weight, regardless of whether the animal developed clinical disease leading to euthanasia or not, indicating that the disease produced by lower doses was insufficient to cause observable weight loss. Marmoset #4 had a 4.2% decrease in body weight between day 3 and day 4 (animal found dead) post-exposure.. The results of the present study are consistent with Adams’ report of weight loss observed in marmosets exposed intranasallyintransally with the EEEV FL93-939 NA strain at 1.0 × 10^6^ PFU [11]. Challenge with the SA strain of EEEV in Adams’ study resembled the results observed for marmoset #2 receiving a lower dose of NA EEEV strain (1.15 × 10^3^ PFU). Because we covered a wider dose range of EEEV doses in our median lethal dose study and possibly due to the use of a different route of exposure, we were able to capture differential effects on weight not previously observed before for the same EEEV strain [11].

Marmosets exposed to the higher doses of EEEV (≥9.76 × 10^4^ PFU) were noted to have developed disease and died roughly 2–3 days following the time of fever onset. This observation is in agreement with Adams’ work as well as with previous data from cynomolgus macaques [11, 17]. Rhesus macaques have also been exposed to EEEV but displayed a more extended survival time following fever onset [27]. The delayed effect on time to death in the rhesus macaque model was also observed in our study, but only for marmosets that were exposed to the lower EEEV doses. These differences in the more prolonged time to death from fever onset for the rhesus may represent a species difference or be a reflection of distinct properties of the viral strains used as well as conditions of viral stock preparation and stability [27]. In the intermediate inhaled doses of EEEV in the marmosets, it was noticed that there was an increase in nocturnal activity for marmoset #2 at day 6 and for marmoset #4 at day 5 (data not shown); again, showing that within the “intermediate” range of inhaled doses, a more clearly traceable pattern of EEEV-driven effects could be detected. Interestingly, effects on blood oxygen saturation were more noticeable in this intermediate range, where weight loss was also considerably affected. A decrease in oxygen saturation levels in the blood has been previously shown in human influenza cases to reflect ongoing inflammatory responses and those results correlated with body weight loss, body temperature changes, and development of lung pathology [28]. The observed depression in oxygen saturation levels in the marmosets may also signal inflammatory responses captured in the animals within these intermediate EEEV aerosol doses.

Although EEEV disease progressed on an altered time course dependent on the aerosol dose, once animals developed clinical disease, the pathological changes were similar between marmosets exposed to increasing inhaled doses with the exception of observed vasculitis. Vasculitis was present in marmosets that received doses greater than 1.0 × 10^4^ PFU, suggesting that it may a dose-related phenomenon. Vasculitis is an important pathological feature of human EEEV disease and factors into the importance of not only characterizing an animal model that will develop vasculitis, but provides insight into the aerosol dose required to mimic the disease course in humans. Therefore, aerosol doses greater than 1.0 × 10^4^ PFU should be considered when using the marmoset as a disease model for aerosolized EEEV. Meningoencephalitis was also noted in our marmosets, and is consistent with autopsy observations made for fatal human EEEV cases [29]. We observed positive antigen staining is the pons and thalamus regions of the brain from several of the marmosets; this observation of involvement of specific areas of the brain is consistent with reports of EEEV-induced signaling anomalies in the basal ganglia, brainstem, and thalamus from magnetic resonance imaging (MRI) in both severe non-fatal and fatal human infections [30–32].

Viremia was not detected in the blood by plaque assay at any time point, but viral genomic RNA was detected by RT-PCR at days 9 and 12 post aerosol exposure in the survivor marmoset receiving the lowest inhaled dose (#1). Expression of EEEV has been shown to be restricted to myeloid cells due to binding of microRNA miR-142-3p at three conserved target sites within the 3′UTR of the EEEV genome, which serves to functionally block viral replication in cell types other than myeloid cells [33, 34]. Overall, our data are in agreement with previous work in marmosets that showed no viremia developed at a high dose with the NA strain of EEEV but viremia was detected when the marmosets were infected intranasally with the SA strain (now re-classified as Madariaga virus) [11]. It is possible that the cell-type constraints found in EEEV due to binding of miR142-3p in a conserved region of the 3′ UTR do not occur in Madariaga virus.

The main hematological change reported for EEEV infection in humans and animal models is leukocytosis late in infection, reflecting in great part the induction of granulocytes [6, 15, 17]. Not only were general increases in white blood cells observed but also increases in absolute numbers of neutrophils, lymphocytes, and monocytes. Interestingly, monocytes have been shown to be refractory to EEEV infection, with a possible exception of an immortalized monocytic cell line pre-treated with a mitogen [35–37]. Outside of the known resistance of monocytes to EEEV infection, these cells, along with lymphocytes, were induced by EEEV in our work, even at non-lethal aerosol doses, underscoring their role in modulating EEEV infection. In addition, a downward trend in the number of platelets was observed after EEEV exposure, although it is not as conclusive for the survivor. The observation of thrombocytopenia in the marmosets that succumbed to disease is in agreement with the human disease course of severe EEEV infection [38]. Platelet depletion has been previously associated with the increasing development of vasculitis and potential coagulopathies. The hematology data from the marmosets therefore describes an early initiated neutrophilic response followed by a lymphocytic response in the acute phase and culminating with monocytosis; this observation was more evident in marmosets receiving lower doses of the virus. Because the high-dosed animals did not live long enough for the body to fully respond with monocytosis, this appears to be a dose-related event, where lower doses may allow the animal to live long enough to mount a robust immune response that includes monocytosis.

Limitations to the study include the small number of animals tested to estimate the median lethal dose; however, the clinical, hematologic, and pathology data are consistent with reports from other nonhuman models of infection and thus support the use of the marmoset as a novel aerosol model of inhalation EEEV for countermeasure efficacy testing. Follow on studies will build upon the work described herein to further refine the median lethal dose with larger numbers of animals to achieve statistical significance, as well as investigate the temporal changes in disease pathology.

## Conclusion

This work has evaluated the clinical and pathological effects of aerosolized EEEV in the common marmoset and estimated the median lethal dose for aerosolized EEEV. The marmoset has now been shown to be a promising animal model for both the intranasal and aerosol routes of EEEV encephalitic disease. We have demonstrated the pathological effects of EEEV disease over a range of doses and describe disease markers that can used with the model for both therapeutic and prophylactic studies. The results demonstrate that the marmoset is an animal model suitable for emulation of human EEEV disease in the development of medical countermeasures.

## Additional file

Additional file 1: Adult Marmoset (*Callithrix jacchus*) hematological and serum chemistry reference values (median and -/+ 3 Standard Deviations): Comparison between locally-established reference intervals intervals. (XLSX 18 kb)
