# Erratum to: Characterization and pathogenesis of aerosolized eastern equine encephalitis in the common marmoset (*Callithrix jacchus*)

**DOI:** 10.1186/s12985-017-0717-5

**Published:** 2017-03-10

**Authors:** Aimee I. Porter, Rebecca A. Erwin-Cohen, Nancy Twenhafel, Taylor Chance, Steven B. Yee, Steven J. Kern, David Norwood, Laurie J. Hartman, Michael D. Parker, Pamela J. Glass, Luis DaSilva

**Affiliations:** 1 Virology Division, United States Army Medical Research Institute of Infectious Diseases (USAMRIID), 21702 Frederick, MD USA; 2 Pathology Division, 21702 Frederick, MD USA; 3 Center for Aerobiological Sciences, 21702 Frederick, MD USA; 4 Research Support Division, 21702 Frederick, MD USA; 5 Diagnostic Systems Division, 21702 Frederick, MD USA

## Erratum

Upon publication of the original article [1], it was noticed that the author's correction requests for Table 2 had not been incorporated, due to an error in the systems. These corrections are listed below and have also been corrected in the original article:

**Table 2:** Viral Plaque Assay and RT-PCR results in solid tissues from marmosets exposed to aerosolized EEEV

Tissue	Animal ID (Inhaled Dose, PFU)									
	marmoset #1 (2.40 x 10^1^)		marmoset #2 (1.15 x 10^3^)		marmoset #3 (1.2 x 10^4^)		marmoset #4 (9.76 x 10^4^)		marmoset #5 (7.95 x 10^5^)	
	Plaque Assay (PFU/g)	PCR	Plaque Assay (PFU/g)	PCR	Plaque Assay (PFU/g)	PCR	Plaque Assay (PFU/g)	PCR	Plaque Assay (PFU/g)	PCR
Salivary Gland	**-**	**-**	**-**	**-**	**1.4E + 04**	**-**	**-**	**-**	**-**	-
Adrenal Gland	**-**	**-**	**-**	**-**	**-**	**-**	**-**	**-**	**1.2E + 04**	+
Pancreas	**-**	**-**	**-**	**-**	**-**	**-**	**-**	**-**	**-**	-
Lung	**-**	**-**	**-**	**-**	**-**	**-**	**-**	**-**	**2.0E + 04**	-
Spleen	**-**	**-**	**-**	**-**	**-**	**+**	**-**	**+**	**-**	-
Axillary LN	**-**	**-**	**-**	**-**	**-**	**-**	**-**	**+**	**-**	-
Kidney	**-**	**-**	**-**	**-**	**-**	**+**	**1.3E + 04**	**+**	**9.5E + 03**	-
Brain	**-**	**-**	**-**	**+**	**1.6E + 07**	**+**	**4.2E + 04**	**+**	**-**	+
Heart	**-**	**-**	**-**	**-**	**-**	**-**	**3.6E + 03**	**-**	**-**	+
Liver	**-**	**-**	**-**	**-**	**-**	**+**	**1.9E + 04**	**-**	**-**	+
Inguinal LN	**-**	**-**	**-**	**-**	**4.2E + 04**	**-**	**-**	**-**	**-**	-
Mandibular LN	**-**	**-**	**-**	**-**	**4.2E + 04**	**+**	**-**	**-**	**7.4E + 03**	-
Tracheo-bronchial LN	**NT**	**NT**	**-**	**-**	**-**	**+**	**5.0E + 04**	**-**	**-**	+
Mesentric LN	**-**	**-**	**-**	**-**	**-**	**-**	**7.3E + 04**	**-**	**-**	+
Popliteal LN	**-**	**-**	**-**	**-**	**-**	**+**	**-**	**-**	**-**	-

*NT* no tissue

+ positive assay result, − = negative assay result

1.) The first column in the body of the table should be labelled "Tissue"

2.) The second column of data should be labelled with the animal # and inhaled dose which it received: "marmoset #1 (2.4 × 10^1)"

3.) There was a misspelling of one of the tissues in the first column: “Trachbronical LN” should be "Tracheobronchial LN"
